# Stress relief may promote the evolution of greater phenotypic plasticity in exotic invasive species: a hypothesis

**DOI:** 10.1002/ece3.1424

**Published:** 2015-02-19

**Authors:** Qiao Q Huang, Xiao Y Pan, Zhi W Fan, Shao L Peng

**Affiliations:** 1 Environment and Plant Protection Institute, Chinese Academy of Tropical Agricultural SciencesHaikou, 571101, China; 2 State Key Laboratory of Biocontrol, School of Life Sciences, Sun Yat-Sen (Zhongshan) UniversityGuangzhou, 510006, China; 3 Institute of Biodiversity Science, Fudan UniversityShanghai, 200433, China

**Keywords:** Adaptive plasticity, costs of plasticity, environmental stress, fitness, invasive species, mechanism of invasion

## Abstract

Invasion ecologists have often found that exotic invaders evolve to be more plastic than conspecific populations from their native range. However, an open question is why some exotic invaders can even evolve to be more plastic given that there may be costs to being plastic. Investigation into the benefits and costs of plasticity suggests that stress may constrain the expression of plasticity (thereby reducing the benefits of plasticity) and exacerbate the costs of plasticity (although this possibility might not be generally applicable). Therefore, evolution of adaptive plasticity is more likely to be constrained in stressful environments. Upon introduction to a new range, exotic species may experience more favorable growth conditions (e.g., because of release from natural enemies). Therefore, we hypothesize that any factors mitigating stress in the introduced range may promote exotic invaders to evolve increased adaptive plasticity by reducing the costs and increasing the benefits of plasticity. Empirical evidence is largely consistent with this hypothesis. This hypothesis contributes to our understanding of why invasive species are often found to be more competitive in a subset of environments. Tests of this hypothesis may not only help us understand what caused increased plasticity in some exotic invaders, but could also tell us if costs (unless very small) are more likely to inhibit the evolution of adaptive plasticity in stressful environments in general.

## Introduction

**Figure 1:**
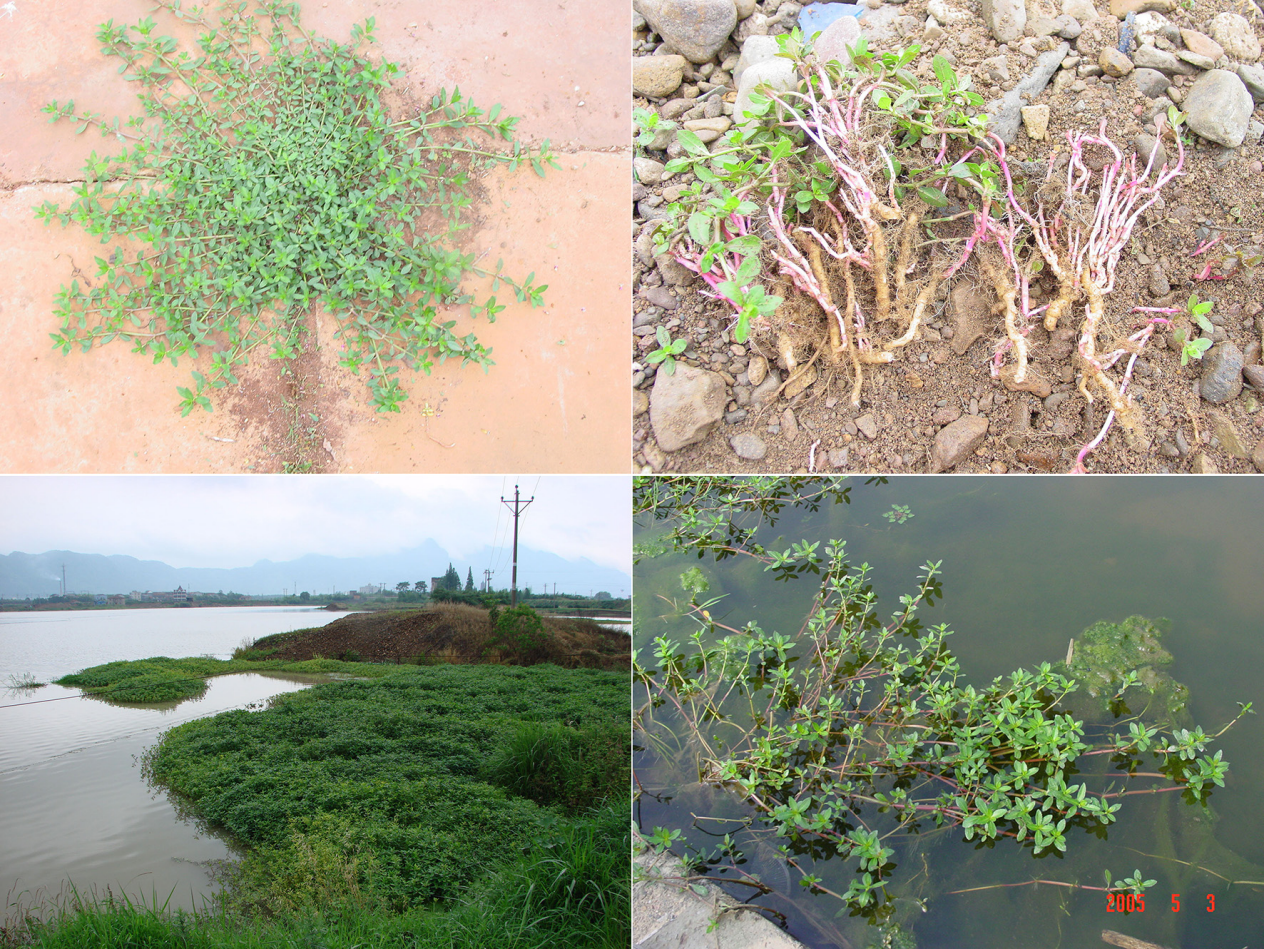
Phenotypic plasticity allows the invasive alligator weed (*Alternanthera philoxeroides* (Mart.) Griseb.) to grow both on land (upper part) and in water (lower part) in China. *A. philoxeroides* allocates more biomass to roots on land than in water. Photograph: Xiao Y. Pan.

Elucidating the mechanisms that facilitate the invasion of exotic species is a fundamental goal of invasion ecology. Phenotypic plasticity, the ability of a genotype to express alternative phenotypes in a range of environments (Bradshaw 1965), is a potential mechanism that has received much attention (Fig.1) (Davidson et al. 2011; Palacio-lópez and Gianoli 2011). While researchers have hypothesized that populations in the introduced range of an invasive species have become more plastic than populations from their native range (Richards et al. 2006), empirical studies have found evidence that both support (Lavergne and Molofsky 2007; Caño et al. 2008) and contradict (DeWalt et al. 2004a; van Kleunen and Fischer 2008) this hypothesis.

In this study, we suggest that these inconsistencies may arise from the costs of plasticity (van Tienderen 1991; Moran 1992; DeWitt et al. 1998; Sultan and Spencer 2002; Callahan et al. 2008; Auld et al. 2010), which determine whether the evolution of increased plasticity is advantageous. By synthesizing the literature on the benefits and costs of plasticity and on exotic species invasions, we propose the hypothesis that relief from stress in the introduced range of a species may promote some exotic invaders to evolve increased phenotypic plasticity by increasing and reducing the benefits and costs of plasticity, respectively. The native-invaded range comparisons (van Kleunen et al. 2010) are important to the working of this hypothesis. Although the comparisons between related native and invasive species may indicate whether invasive species are more plastic than co-occurring natives, demonstrating the evolution of plasticity in invasive species requires comparisons between native and invasive populations of the same species (van Kleunen et al. 2010). In this study, we will mostly discuss plants, but our argument is widely applicable to other taxa as well.

For the evolution of increased plasticity to be adaptive, there needs to be a link between the plasticity of traits and fitness. Generally, natural selection will act to increase the fitness of populations across environments, which is often achieved through plasticity in underlying morphological, physiological, behavioral, and growth traits that influence fitness (Richards et al. 2006). Trait plasticity may allow species to achieve a consistent level of fitness between individuals across a range of environments. Therefore, plasticity in underlying functional traits may potentially increase or maintain high levels of fitness. Ultimately, what matters for the invasion success of exotics is the fitness consequences of the evolution of greater plasticity.

Although a plastic response to environmental variation is often thought to be beneficial, such a response cannot necessarily be assumed to be adaptive. Neutral or maladaptive plasticity can result from reduced fitness as a consequence of resource limitation, unpredictable changes in environments or unreliable cues, pleiotropy, and genetic drift (van Kleunen and Fischer 2005). Adaptive plasticity requires that plasticity in functional traits enhances fitness (Scheiner 1993). However, a general consensus on the adaptive significance of plasticity exists for just a few traits, such as elongation of plant internodes and increased specific leaf area in response to shading (Schmitt et al. 1995), and induced defenses to herbivores, predators, and pathogens (Agrawal 1998). For many other traits in which plasticity has been assumed to be adaptive, the evolution of increased plasticity in these traits needs to be shown to increase individual fitness.

## Development of the Hypothesis: Linking Phenotypic Plasticity of Invasive Species to Its Potential Costs

### Stress relief and exotic species invasions

Stress in this study refers to herbivory and poor growth conditions due to competition or low resource availability. Invasive species are those that establish and spread after being introduced to a novel range. Upon introduction to a new range, exotic species may be released from natural enemies. The natural enemy release hypothesis (Keane and Crawley 2002) states that exotic species leave behind specialist enemies when introduced, thereby alleviating stressful growth conditions because of the reduced abundance of natural enemies. In support of this hypothesis, many studies have found that exotic invasive plants and animals are released from natural enemies (e.g., Porter et al. 1997; DeWalt et al. 2004b) and that more invasive species experience enemy release more strongly than less invasive species (Mitchell and Power 2003). However, we also note that not all studies found support for the natural enemy release and that some exotics may be attacked more by novel natural enemies in the introduced range (Verhoeven et al. 2009). Exotic species may also increase their competitive ability and resource availability through the use of novel weapons (Callaway and Aschehoug 2000) and through positive feedback with soil biota (Callaway et al. 2004), although some may invade resource-poor and competitive environments and may thus not experience stress relief (Levine et al. 2004; Drenovsky et al. 2012). Therefore, although there are exceptions, there are at least a portion of exotic invaders that experience stress relief in the introduced range, and more invasive species may experience a stronger stress relief than less invasive species.

### Benefits and costs of plasticity in relation to environmental stress

We first explain what we mean by the benefits and costs of plasticity and under what conditions the evolution of greater plasticity will be inhibited. We assume that there is a plastic genotype and a nonplastic genotype of a species encountering a range of heterogeneous environments *E*_1_, *E*_2_, … and *E*_*n*_ (e.g., a range of light levels) with their frequencies being *r*_1_, *r*_2_, … and *r*_*n*_ (0 ≤ *r*_j _< 1 and 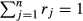 ), respectively. The nonplastic genotype did not change its phenotype, but the plastic genotype may change its phenotype as environments change. With all else being equal, the plastic genotype may experience a fitness reduction under *E*_*k*_ (with the value being *c*_*k*_, *c*_*k *_≥ 0; 1 ≤ *k* < *n*), but a fitness increase under *E*_*k+m*_ (with the value being *b*_*k+m*_, *b*_*k+m *_> 0; 1 ≤ *m* ≤ *n* – *k*) compared with the nonplastic genotype. The fitness increase of the plastic genotype under *E*_*k+m*_ is the benefit of plasticity, which arises because a fitter phenotype is expressed. The reduction in fitness of the plastic genotype under *E*_*k*_ is the cost of plasticity, which may reflect the resources allocated for maintaining the ability to be plastic (van Tienderen 1991; DeWitt et al. 1998; Auld et al. 2010). Such a definition of costs of plasticity is somewhat different from that of DeWitt et al. (1998) and van Kleunen and Fischer (2005) because costs in this definition may be caused by the limits of plasticity (i.e., the plastic genotype cannot attain the same trait value enhancing fitness in the focal environment compared with the nonplastic genotype). However, our definition is consistent with that used by several other authors (Moran 1992; Auld et al. 2010). The evolution of increased plasticity will be inhibited when the global fitness of the plastic genotype is smaller than the global fitness of the nonplastic genotype (Moran 1992):

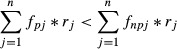
1

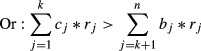
2

where *f*_*pj*_ and *f*_*npj*_ are the fitnesses of the plastic genotype and the nonplastic genotype under *E*_*j*_, respectively, and k is the number of environments where costs of plasticity exist.

The key assumption in this section is that the evolution of greater adaptive plasticity is more likely to be inhibited in stressful environments. This conclusion requires that two basic assumptions to be met: (1) costs of plasticity should be more severe under stress, and (2) the benefits of plasticity may be reduced under stress. Reviews on plants and animals suggested that stress may inhibit the expression of plasticity, either because the potential plastic response in a given trait cannot be fully achieved due to resource limitation, or because expressing plasticity would make organisms more vulnerable to stress (Valladares et al. 2007; Steinberg 2012). For example, in 19 of 24 cases, damage or herbivore attacks reduced the plastic response of plants to changing abiotic conditions (Valladares et al. 2007). This finding suggests that in most cases, damaged plants could not attain an optimal phenotype in the challenging environment; thus, the benefits of plasticity may be reduced. However, we note that this point may not be general and that there may be many cases where plasticity is still useful under stressful conditions even if benefits of plasticity are reduced.

Costs of plasticity have proven rather elusive because most empirical studies failed to detect such costs (van Kleunen and Fischer 2005; van Buskirk and Steiner 2009). A potential reason is that costs may vary in magnitude depending on environmental conditions. Several studies suggest that costs of plasticity are more easily detected under stress (Dorn et al. 2000; Stinchcombe et al. 2004; Weinig et al. 2006). For example, genotypes of *Ranunculus reptans* L. that were more plastic in internode length in response to competition produced on average fewer rosettes and flowers relative to less plastic genotypes with the same internode length. However, this difference was only detected when plants were grown in environments with competitors (van Kleunen et al. 2000). Similar results were also observed in *Impatiens capensis* Meerb. (Donohue et al. 2000) and *Sinapsis arvensis* L. (Steinger et al. 2003). These studies only found costs of plasticity in more stressful test environments. This finding suggests that plastic traits can induce larger fitness costs for organisms living in stressful environments than for those living in favorable ones. Under stress, organisms may show fixed development rather than plasticity (Chapin 1980; Grime et al. 1986; Balaguer et al. 2001), although there is also much evidence suggesting that species in stressful and favorable environments are equally plastic (Fransen et al. 1998; Alpert and Simms 2002).

The reason for the point that costs of plasticity are more severe under stress may be that when resources are limited, allocating resources for maintaining the ability to be plastic can have a larger negative impact on fitness than when resources are not limited. However, a meta-analysis has found that this point has only been supported in studies of animals, but not in studies of plants (van Buskirk and Steiner 2009). One possible reason may be that most studies evaluating costs of plasticity are not conducted under natural conditions where resources are limited, but in glasshouse or growth-chamber environments that are still favorable even under more stressful treatments (Weinig et al. 2006; Dechaine et al. 2007). Overall, because of the current difficulty in detecting costs of plasticity and limited research in this area (van Buskirk and Steiner 2009; Auld et al. 2010), we do not know to what extent this point reflects general patterns in nature. Nevertheless, it appears to be important in some situations.

### The hypothesis and its predictions

Our hypothesis states that stress relief in the introduced range may promote some exotic invaders to evolve greater phenotypic plasticity by reducing the costs and increasing the benefits of plasticity, and it is derived from a combination of the above two conceptual fields that some exotic species may be released from stress in the introduced range and that benefits of plasticity may be lower and costs of plasticity may be more severe under stress. Some preconditions are necessary for this hypothesis. First, despite differences in the stressfulness of growth conditions between native and introduced ranges (e.g., the extent of herbivory, which should differ from the environmental variable that induces a plastic response), the plastic trait under selection must be adaptive (i.e., there are benefits of plasticity at least under a portion of environments) for the invasive populations, although this may not be the case for native populations. A plastic trait that is adaptive under some growth conditions may not be adaptive under other growth conditions. For example, under release from natural enemies, plasticity in competition-avoidance traits (e.g., internode elongation) may no longer be necessary because the exotics have additional resources to outcompete neighboring plants. Our hypothesis does not apply to such situations. However, some neutral or maladaptive plasticity in stressful environments may become adaptive in favorable environments. Our hypothesis could apply to such situations (in such situations, the benefits of plasticity are zero or a negative value in stressful environments). Second, there must be genetic variation in plasticity. Actually, there may be cases where a lack of genetic variation in plasticity limits plasticity evolution. However, although genetic variation may be limited for some invasive species, in general, levels of genetic variation in invasive species are high (Lavergne and Molofsky 2007; Roman and Darling 2007; Verhoeven et al. 2011).

We assume that in the introduced range, invasive populations encounter a range of heterogeneous environments (e.g., a range of light levels) *E*_1_, *E*_2_, … and *E*_*n*_ with their frequencies being *r*_*i*1_, *r*_*i*2_, … and *r*_*in*_ (0 ≤ *r*_*ij *_< 1 and 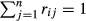), respectively, and that in the native range, native populations of the species encounter the same set of environments *E*_1_, *E*_2_, … and *E*_*n*_ with their frequencies being *r*_*n*1_, *r*_*n*2_, … and *r*_*nn*_ (0 ≤ *r*_*nj *_< 1 and 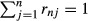), respectively. We also assume that invasive populations experience stress relief such as natural enemy release. Following equation 2, if stress relief promotes the evolution of increased phenotypic plasticity in the introduced range by reducing the costs of plasticity and increasing the benefits of plasticity, the following two conditions need to be met:

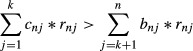
3

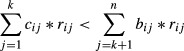
4

where *c*_*nj*_ and *c*_*ij*_ are costs of plasticity under *E*_*j*_ in the native and introduced ranges (*c*_*nj *_> *c*_*ij *_≥ 0), respectively, *b*_*nj*_ and *b*_*ij*_ are benefits of plasticity under *E*_*j*_ in the native and introduced ranges (*b*_*ij*_ > *b*_*nj*_ and *b*_*ij*_ > 0), respectively, and *k* is the number of environments where there are costs of plasticity. In other words, large costs and small benefits of plasticity constrain the evolution of greater plasticity in the native range, but reduced costs and increased benefits of plasticity promote the evolution of greater plasticity in the introduced range. Additionally, a larger 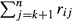 than 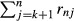 (i.e., invasive populations more frequently encounter environments where there are benefits of plasticity) will promote the evolution of greater plasticity in the introduced range.

**Figure 2:**
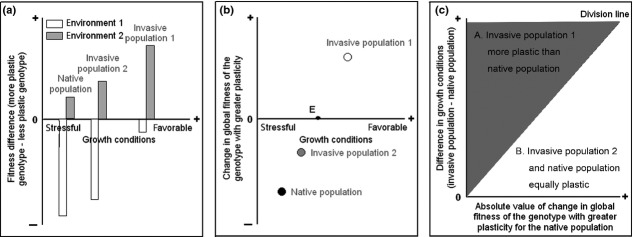
A hypothetical example showing how stress relief in the introduced range may promote the evolution of greater plasticity in an exotic invader by reducing the costs and increasing the benefits of plasticity. The environmental variable that determines stress level in the native and introduced ranges (the *x*-axis in (a) and (b); e.g., extent of natural enemy attack) should be different from heterogeneous environments 1 and 2 (e.g., high and low light levels) that induce a plastic response, and the growth conditions are more stressful in the native range than in the introduced range. The frequencies of the two environments 1 and 2 in the introduced range are *r*_*i*1_ and *r*_*i*2_, respectively, and the frequencies of the same two environments 1 and 2 in the native range are *r*_*n*1_ and *r*_*n*2_, respectively. Change in global fitness (i.e., the fitness averaged across the two environments) of the genotype with greater plasticity is calculated as the global fitness of the more plastic genotype minus the global fitness of the less plastic genotype, with + and – indicating an increase and a reduction, respectively. Values chosen: *r*_*i*1_ = *r*_*i*2_ = *r*_*n*1_ = *r*_*n*2_ = 0.5. (a) Fitness difference between more plastic and less plastic genotypes for invasive and native populations under alternative environments 1 and 2, (b) change in global fitness of the genotype with greater plasticity for invasive and native populations, and (c) the outcome of relative plasticity between invasive and native populations. In (a), the fitness difference between more plastic and less plastic genotypes under environment 2 is the benefit of plasticity, and the absolute value of the fitness difference between more plastic and less plastic genotypes under environment 1 is the cost of plasticity. In (b), the point E is located on the *x*-axis, and it represents the growth conditions under which more plastic and less plastic genotypes do not differ in global fitness. The native population is located below point E, the invasive population 2 is located between point E and the native population, and the invasive population 1 is located above point E. The line that divides regions A and B in (c) is the point E in (b).

Figure2 displays the plasticity scenarios using a two-state environmental variable (e.g., high and low light levels). From stressful to favorable growth conditions (i.e., from native to introduce ranges), costs of plasticity decrease and benefits of plasticity increase (Fig.2a). For invasive population 1, the benefits can offset the costs of plasticity, and with greater plasticity, invasive population 1 can increase its global fitness averaged over environments (Fig.2b). The outcome is that invasive population 1 evolves to be more plastic than the native population (region A in Fig.2c). For invasive population 2, the benefits of plasticity do not offset its costs despite experiencing more favorable growth conditions than the native population, and with greater plasticity, the global fitness of the invasive population 2 would decrease (Fig.2b). The outcome is that invasive population 2 and the native population are both equally plastic (region B in Fig.2c).

The hypothesis emphasizes that differences in growth conditions between invasive and native populations can have diverse effects on the outcomes of invasion, modulated by how the evolution of increased plasticity is inhibited by its costs in native populations growing in more stressful conditions. The hypothesis formalizes several points that help resolve confusion over species plasticity and invasion. First, our hypothesis predicts that, under stress, a large reduction in global fitness for genotypes with more plastic traits increases the chance that invasive and native populations will exhibit the same level of plasticity. Second, more favorable growth conditions for the invader than for the native increase the probability that the invader is more plastic than the native. Thus, our hypothesis predicts that greater plasticity is most likely for highly successful invaders that experience more favorable growth conditions. Third, integrating the first two points, greater plasticity in exotic invasive species requires a small reduction in global fitness for native populations that constrains their evolution of greater plasticity despite less stressful growth conditions experienced by invasive populations. For example, invasive populations should be released from natural enemies and can then easily afford the costs that native populations cannot afford, thus evolving greater plasticity.

### Empirical studies on phenotypic plasticity of invasive plants

Research on phenotypic plasticity of exotic invasive plants has found that exotic invasive populations have evolved to be more or equally plastic than conspecific populations from their native range (Bossdorf et al. 2005; Richards et al. 2006; Matesanz et al. 2010). These studies focused on exotic invaders that were dominant, highly successful, released from natural enemies, or at least common, indicating that they probably experienced more favorable growth conditions. Therefore, these results are largely consistent with the prediction that stress relief may, but not always, facilitate an exotic invader to evolve greater adaptive plasticity, although we should note that it is not known whether equal plasticity between invasive and native populations arises because costs still inhibit the evolution of greater plasticity in invasive populations (invasive population 2 in Fig.2), or because of other unknown reasons.

## Implications and Future Research Needs

### Implications of the hypothesis

A frequently proposed hypothesis explaining greater adaptive plasticity in some exotic invaders is that adaptive plasticity may be necessary for invasion. Among alien species or several genotypes of one species, those that have greater adaptive plasticity are more likely to establish and become invasive (van Kleunen et al. 2011). This explanation stresses that exotics may become invasive because of exaptation, and it does not require any assumption about costs of plasticity. Our hypothesis brings a new explanation as to why some exotic invaders can be more plastic based on benefit–cost analyses, and it mainly accounts for the postinvasion evolution of plasticity (e.g., Lavergne and Molofsky 2007). These hypotheses and explanations together could explain the greater plasticity among exotic invaders.

Our hypothesis helps understand why exotic invaders are often found to be more competitive only in a subset of environments (Daehler 2003). If an invader evolves to be more plastic, its fitness and competitive ability will increase in one particular set of environments (benefit from greater plasticity), but may not change in another (the effects of induced costs and alleviated environmental stress may cancel out). This expectation also implies that the resources saved from enemy defense may be reallocated to increase both plasticity and competitive ability (Blossey and Nötzold 1995; Bossdorf et al. 2005). Our hypothesis can thus be recognized as an extension of the evolution of increased competitive ability (EICA) hypothesis (Blossey and Nötzold 1995) because plasticity is not the same as competitive ability, but our hypothesis differs from the EICA hypothesis in two aspects. First, the EICA hypothesis requires that there are resources allocated to defense in native populations of an exotic species. However, resources saved from a reduction in herbivory – in addition to resources saved from enemy defense – can promote the evolution of greater adaptive plasticity. Second, increased competitive ability and resource availability due to novel weapons (Callaway and Aschehoug 2000) may also promote the evolution of greater adaptive plasticity (e.g., increased induced defense, Cipollini et al. 2005).

Our hypothesis also indicates that future environmental changes (e.g., increased human activities and climate change) may promote biological invasions. Under environmental changes, the environmental conditions will be more heterogeneous, and a part of exotic species that are better able to show evolution in the plasticity may have a higher probability to colonize and invade.

Finally, our hypothesis implies that organisms in favorable environments can be either more or equally plastic than those in stressful conditions. Equal plasticity is either because organisms in the contrasting environments can all be plastic as costs of plasticity are very small, or because costs inhibit the evolution of plasticity in organisms living in both favorable and stressful environments.

### Tests of the hypothesis

Although research on phenotypic plasticity of invasive species is consistent with our hypothesis, this evidence is not direct and sufficient. Here, we propose ways to directly test the hypothesis.

We assume that the growth conditions of exotic invaders are more favorable in the introduced range than in the native range. To test the hypothesis, one should first test whether invasive populations are more plastic in some functional traits than native populations under a range of heterogeneous environments *E*_1_, *E*_2_, … and *E*_*n*_. If the invasive populations show greater plasticity, one should determine whether this plasticity is adaptive. One should compare the fitnesses of invasive populations (i.e., more plastic genotypes) and native populations (i.e., less plastic genotypes) under a range of heterogeneous environments *E*_1_, *E*_2_, … and *E*_*n*_ in both the introduced and native ranges. After measuring the frequency of each environment in both the introduced and native ranges, one can examine whether equations 3 and 4 are met. If so, the hypothesis is strongly supported.

If natural enemy release explains the more favorable growth conditions in the introduced range, one can use herbivore exclusion treatments in the native range as a substitute for natural enemy release in the introduced range because it may not be very feasible to grow organisms from invasive and native populations in both the introduced and native ranges.

If it is also difficult to measure the environmental heterogeneity factor *r*, one can only compare the fitnesses of invasive populations (i.e., more plastic genotypes) and native populations (i.e., less plastic genotypes) under a range of heterogeneous environments *E*_1_, *E*_2_, … and *E*_*n*_ in both stressful (i.e., simulating those in the native range) and favorable growth conditions (i.e., simulating those in the introduced range). Without measuring the environmental heterogeneity factor *r*, one can examine whether costs of plasticity are more severe under stress and benefits of plasticity are lower under stress (i.e., two basic assumptions of the hypothesis).

Ideally in these tests, one should study fast-growing species adapted to high resource availability, to make sure there is no evolution toward reduced defenses and increased growth in invasive populations (Blossey and Nötzold 1995; Blumenthal 2006; Pan et al. 2012). Otherwise, the performance of invasive populations may be confounded by factors such as reduced defenses or increased growth (e.g., more natural enemy attack on invasive populations because of reduced defenses when they are grown in the native range).

## Conclusions

The role of phenotypic plasticity in exotic species invasions is a hot topic in invasion ecology. However, currently, no hypothesis has been established to explain where and why exotic species might evolve greater plasticity after their introduction. By synthesizing the literature on the benefits and costs of plasticity and that on exotic species invasions, we propose the hypothesis that natural enemy release or any other factor inducing the relief of stress in the introduced range may promote some exotic invaders to evolve greater adaptive plasticity by reducing the costs and increasing the benefits of plasticity. The hypothesis is largely consistent with empirical findings. The hypothesis implies that different mechanisms of invasion may be interrelated and brings a series of new insights to the understanding of biological invasions and costs of plasticity (in cases where plasticity is in fact costly). Finally, the hypothesis indicates that stress relief in the introduced range plays a fundamental role in driving invasions by directly promoting invasiveness and indirectly facilitating the evolution of invasiveness in exotic species, further enhancing their invasion potential.
